# Trivially informative semantic context inflates people's confidence they can perform a highly complex skill

**DOI:** 10.1098/rsos.211977

**Published:** 2022-03-16

**Authors:** Kayla Jordan, Rachel Zajac, Daniel Bernstein, Chaitanya Joshi, Maryanne Garry

**Affiliations:** ^1^ School of Psychology, The University of Waikato, 1 Knighton Road, Hamilton 3240, New Zealand; ^2^ School of Psychology, University of Otago, 362 Leith Street, Dunedin 9016, New Zealand; ^3^ Department of Psychology, Kwantlen Polytechnic University, 12666 72 Ave, Surrey, British Columbia V3W2M8, Canada

**Keywords:** overconfidence, fluency, semantic context

## Abstract

Some research suggests people are overconfident because of personality characteristics, lack of insight, or because overconfidence is beneficial in its own right. But other research fits with the possibility that fluent experience in the moment can rapidly drive overconfidence. For example, fluency can push people to become overconfident in their ability to throw a dart, know how rainbows form or predict the future value of a commodity. But surely there are limits to overconfidence. That is, even in the face of fluency manipulations known to increase feelings of confidence, reasonable people would reject the thought that they, for example, might be able to land a plane in an emergency. To address this question, we conducted two experiments comprising a total of 780 people. We asked some people (but not others) to watch a trivially informative video of a pilot landing a plane before they rated their confidence in their own ability to land a plane. We found watching the video inflated people's confidence that they could land a plane. Our findings extend prior work by suggesting that increased semantic context creates illusions not just of prior experience or understanding—but also of the ability to actually do something implausible.

## 1. Introduction

Imagine this: you're on a small commuter plane when the pilot becomes incapacitated. You are the only other person on the plane. How confident are you that you could land the plane without dying? It seems obvious that for anyone without flight experience that confidence should be vanishingly small. After all, landing a plane is a highly specialized skill, requiring hundreds of hours of training and a deep understanding of physics, engineering and meteorology [1,2]. But here we present evidence that a disturbing proportion of ordinary people display some confidence they could land a plane safely, and that they become even more confident after we show them one trivially relevant piece of information: a 3 min video of a pilot landing a plane.

Several literatures help us to see how this effect might emerge. One literature addresses the degree to which people are overconfident on the basis of their own characteristics. Take one type of overconfidence in this literature, called ‘overclaiming’—or claiming to know terms that do not exist. Overclaiming is thought to be driven by social desirability, the need for self-enhancement, or even narcissism [3,4]. In other words, the idea is that people **‘**overclaim’ what they know, or what they can do, so other people think better of them [5].

Another kind of overconfidence is the ‘Dunning Kruger effect’. Across a wide range of skills— including humour, grammar and logic—the worst performers overestimate their abilities the most [6,7]. The effect is said to be driven by a metacognitive failure whereby the least skilled people are ‘ignorant of their own ignorance’ [8,9]. Not only can they not execute the target skills effectively, but they also lack the ability to evaluate their own level of skill accurately [8,10].

A third kind of overconfidence, ‘above average’ effects, suggest that most people are prone to making miscalibrated judgements about their knowledge or ability, particularly when comparing themselves with the ‘average’ person [11]. And so, most drivers think they're better than the average driver, most teachers think they're better than the average teacher, and over a third of software engineers think they are among the very top employees in their companies. Obviously, not all of these people can be correct [12–14].

Finally, overconfidence is associated with gender [15,16]. Men tend to be more overconfident in their knowledge and abilities than women—even in a high-stakes environment, such as competitive running and diving [17,18]. This gender overconfidence gap is most prevalent when people are asked to evaluate their performance on a masculine-gender-typed task. By contrast, women do not show the same overconfidence for feminine-gender-typed tasks [16].

These explanations suggest overconfidence arises as some by-product associated with certain characteristics of the person, such as personality traits or lack of insight. But a second literature suggests that overconfidence is beneficial in its own right. We know from the literature on social cognition that believing that we are above average or holding unrealistically positive evaluations about ourselves is a key part of the ‘positive illusions’ most of us have [19]. For example, when people are overconfident in their abilities to do something, it can bias them to focus on the expected benefits rather than the costs [20,21]. This focus on benefits might in turn drive people to pursue more difficult goals, to put in more effort and persistence to accomplish those goals, and sometimes even to accomplish them [19,20]. In fact, overconfident CEOs who invest in risky projects have also been found to be better innovators [22]. There are also social benefits to overconfidence. For instance, people think that overconfident others are more knowledgeable and more trustworthy than their equally competent—but not overconfident—peers, awarding them higher status accordingly [23–25]. Considered together, these literatures suggest that a little self-enhancement does not hurt in most cases, and in fact there are often numerous benefits [20].

Considered together, the research fits with the idea that overconfidence arises from some characteristics about the person, or because overconfidence is beneficial in its own right. But these explanations for overconfidence ignore what's going on in the moment when people assess their confidence. And there is reason to think that experience in the moment can rapidly drive overconfidence in one's knowledge about the world, performance or even their future.

In one line of work, people's tendency to think they understood complex processes more than they actually did was made worse when the to-be-explained process was accompanied by a semantically related but uninformative photo [26]. In another line of work, people said certain recipes were easier to follow, and exercise routines easier to do, when the information was presented in an easy-to-read font than in a difficult-to-read font [27]. Finally, in another study, people who watched demonstrations of magic tricks or dart-throwing 20 times become more confident they could do those same tasks compared with people who watched the demonstrations only once [28].

Collectively, these lines of research fit with the vast literature suggesting that people treat their feelings as information when making judgements about what they know, like, believe and understand [29]. The idea is that when we process information, we are influenced by how easy or difficult it feels to do it [30]. We tend to interpret easy processing, or *fluency*, along positive dimensions, such as truth, ease, attractiveness or evidence of understanding [31–35]. In one study, people judged claims that certain commodities would increase in price as more true when those claims were paired with a semantically related—but ultimately uninformative—photo, than with an unrelated photo or no photo at all [36]. Likewise, work from educational psychology shows that students reported a greater understanding of the scientific underpinnings of climate change when they read a paragraph accompanied by a semantically related but uninformative photo (a flooded street) than when they read the paragraph alone [37]. Studies like these suggest photos might provide a semantic context that increases fluency and makes it easier for people to bring related information to mind. As a result, people might attribute some meaning to the photo due to ease of processing, even though it provided no useful information to inform their judgement [38,39]. This feeling of fluency, in particular conceptual fluency, could in turn be interpreted as evidence of understanding. Taken together, these findings suggest that semantic context can help scaffold new information, connect it to prior knowledge and improve comprehension [40,41]. But in accomplishing these feats, semantic context can backfire, creating misplaced confidence.

One likely explanation for this misplaced confidence is a source monitoring error, whereby the photos make it easier for people to bring to mind thoughts and feelings, with greater vividness and detail, that they construe as evidence that the claim is true [36,42,43]. The Source Monitoring Framework also provides a rationale for people making similar errors in evaluations about the future [44]. A classic paper suggests that both the past and future are ‘constructions of the present’, with the past constantly changing and the future constrained by what we are doing right now [45]. These ideas fit with modern evidence suggesting that imagination and memory are ‘fundamentally the same process' [46]. Indeed, we know people rely on similar characteristics when judging the past and when estimating the likelihood of some event in the future [42,47–50]. Just like their past counterparts, future events are rated more likely when those events come to mind easily, are full of sensory detail and low on cognitive markers of effort [45,51].

But surely there are limits to overconfidence. There is a vast difference between landing a plane and assessing confidence in one's ability to cook a dish, throw a dart, know how rainbows form or predict the future value of a commodity. When it comes to appraising one's highly specialized skills, such as landing a plane, perhaps people can take more reliable shortcuts than turning to feelings as information. Landing a plane is widely known to require expertise that arises from dedicated training and practice. It seems reasonable to speculate that—even in the face of fluency manipulations known to increase feelings of confidence—people might find this fact easily accessible, resulting in more realistic estimates of ability.

Nonetheless, some real-world survey data suggest that people, especially men, can be remarkably overconfident in their ability to do ridiculous things. Data from a recent YouGov survey showed 12% of men claimed they could win a point in a game against 23-time tennis grand slam winner Serena Williams [52,53]. Only 3% of women made this claim. And lest we think this disparity is a single cherry-picked example from the competitive world of sports, in another YouGov survey asking men and women to identify which animals they could beat in a fight, more men than women claimed they could beat every animal. This list included king cobras, bears and eagles [54]. In short, these preposterous examples suggest overconfidence might emerge as well when people are asked if they could land a plane in an emergency. Such a prediction is supported more broadly by a growing experimental literature that suggests that even when people can demonstrate their knowledge of something, they do not always apply that knowledge [55]. In a classic example, people first take a test of general knowledge, and then—in a follow-up session—take a new test. This time, they are told to answer the questions only when they contain correct information [56]. But many people fail to detect errors and mistakenly answer a question such as **‘**how many animals of each kind did Moses take on the ark?’ when their answers on the first test showed they knew Noah was the correct biblical reference [56]. These findings suggest that even in the face of genuine awareness that landing a plane requires expertise, people might still fail to retrieve and apply that awareness in certain situations.

Considered together, several literatures converge on the possibility that, if we increase semantic context by showing people a related but uninformative video of a pilot landing a plane, they could come to rapidly display increased confidence in their ability to land a plane safely. These findings would suggest significant boundary conditions on prior demonstrations. Here, we report a series of experiments to investigate this possibility. We asked some people to watch a video of a pilot landing a plane. Others watched no video. Immediately afterwards, everyone rated their confidence in their own ability to land a plane to a high standard (as well as a pilot could) and to a lower standard (without dying).

## 2. Experiment 1

### 2.1. Method

The materials and data for this experiment are available at https://researchbox.org/511.

#### 2.1.1. Subjects

We recruited subjects from Amazon's Mechanical Turk (MTurk), an online source of a vast and diverse population; subjects received $0.30 USD for participating [57]. In the absence of data to estimate the true size of an effect, we instead aimed to collect data from 100 subjects in each condition. Anticipating exclusions, we calculated data from 227 and retained a total of 198 subjects after exclusions (*M*_age_ = 40.47, s.d._age_ = 13.42; 37% identified as men, 62% identified as women, and 1% identified as gender diverse); 98% of subjects reported English was their first language.

#### 2.1.2. Design

We used a between-subjects design with two conditions (video: video, no video).

#### 2.1.3. Procedure

The experiment proceeded in four phases. First, subjects read the following instruction: ‘Imagine you are on a small commuter plane. Due to an emergency, the pilot is incapacitated and you are the only person left to land the plane’. Second, subjects were randomly assigned either to watch a video of a pilot landing a plane, or to watch no such video. This video was 3 m 44 s and had no audio track. It was filmed from the back of the flight deck and depicted the view of the flight deck, and the back of the pilots. Although it was clear the pilots were engaging with the instruments and controls, views of their hands and what they were doing were obstructed. In short, the video did not teach people how to land a plane and had little instructional merit; in fact, when we showed the video to a retired pilot who flew for over 35 years with Air New Zealand and taught other pilots, he pronounced the video ‘100% useless’. In line with prior work on ‘truthiness’, we therefore classified the video as ‘non-probative’—it was related to the task at hand, but did not teach anyone how to do the task [26,36].

In the third phase, subjects were told:

Now we're going to ask you a few questions. Don't try to analyze and puzzle things out—just go with your gut feel or hunch. Respond as quickly as possible within a couple of seconds. Remember this is an emergency situation.

Then, everyone answered the following confidence questions, in a fixed order because for our first attempt to answer our research question, we aimed to maximize the size of effects by presenting the ‘low bar’ question first [58,59]: ‘How confident are you that you would be able to land the plane without dying’ (0 = *Not at all confident*, 100 = *Very confident*) and ‘How confident are you that you would be able to successfully land the plane as well as a pilot could’ (0 = not at all confident, 100 = very confident). We then asked subjects a series of questions comprising criteria for exclusion (‘Are you a pilot?’ (*Yes, No*), ‘Have you flown a plane before?’ (*Yes, No*) and ‘Have you ever landed a plane before?’ (*Yes, No*)); exploratory investigations of mechanism (‘How difficult was it for you to imagine attempting to land the plane’ (1 = *Not at all difficult*, 5 = *Very difficult*) and ‘How much expertise do you think is involved in landing a plane’ (1 = *No expertise*, 5 = *A great deal of expertise*)); and checks for attention ('Which of the following best describes the situation you were asked to imagine?')

Subjects who watched the video answered these questions immediately after it ended; subjects who did not watch the video answered these questions immediately after the instruction to imagine the scenario. Because we were interested in people's quick, ‘gut feel or hunch’, subjects who did not see the video answered these questions immediately, rather than after a delay matched to the duration of the video.

### 2.2. Results and discussion

Recall our primary research question was: to what extent does watching a video of an expert performing a skill inflate people's confidence in their own ability to perform the skill? Before answering this question, we first excluded 27 subjects who provided non-sensical descriptions of the situation they were asked to imagine, failed our attention check, had a valid pilot's licence, or had flown or landed a plane before. We therefore retained 198 subjects in the final dataset.

We now return to our primary question. To answer this question, we first classified subjects' confidence ratings for the lower standard ‘without dying’ question according to whether they had or had not seen the video.

**Figure 1.**
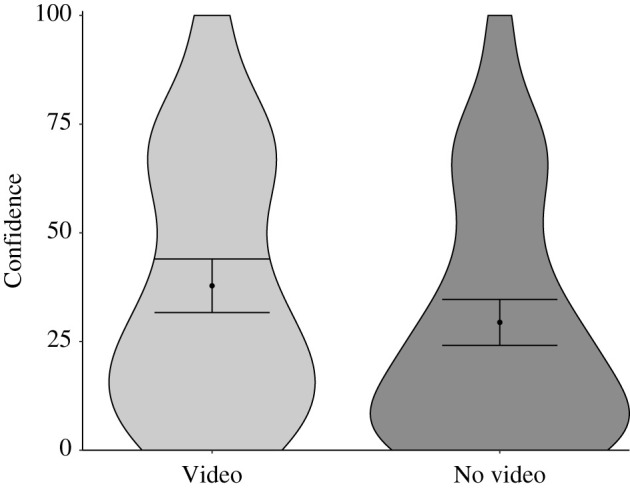
Subjects’ confidence ratings for the lower standard ‘without dying’ question by condition: video, no video in Experiment 1. Error bars represent 95% confidence intervals of the cell means.

We observed a marked skew in confidence ratings: the lower quartile was 9 and the modal response was 0; clearly, no transformation could restore normality. But t-tests—especially Welch's, a parametric test that does not assume equal variance—are robust to most deviations in normality [60,61]. Therefore, we conducted a Welch's t-test. We also conducted a Wilcoxon signed-rank test, a non-parametric test. Both analyses revealed the same pattern, and we display these findings in figure 1. The figure shows two important findings. First, subjects who had watched the video were more confident they could land the plane without dying than those who had not, *M*_video_ = 37.82, *M*_no video_ = 29.41, *M*_diff_ = 8.41, 95% CI [0.35, 16.46]. In null hypothesis testing terms, both the Welch's t-test and Wilcoxon signed-rank test showed the same pattern, *t*_184_ = 2.057, *p* = 0.04; *Z* = 2.15, *p* = 0.03. Second, we asked a pilot with 35 years' experience flying commercially and internationally for Air New Zealand for his estimate on the chances of surviving the landing and his estimate was 10–15%. A one-sample t-test showed that subjects’ responses to the ‘without dying’ question were significantly higher than this estimate, *t*_184_ = 8.92, *p* < 0.001.

**Figure 2.**
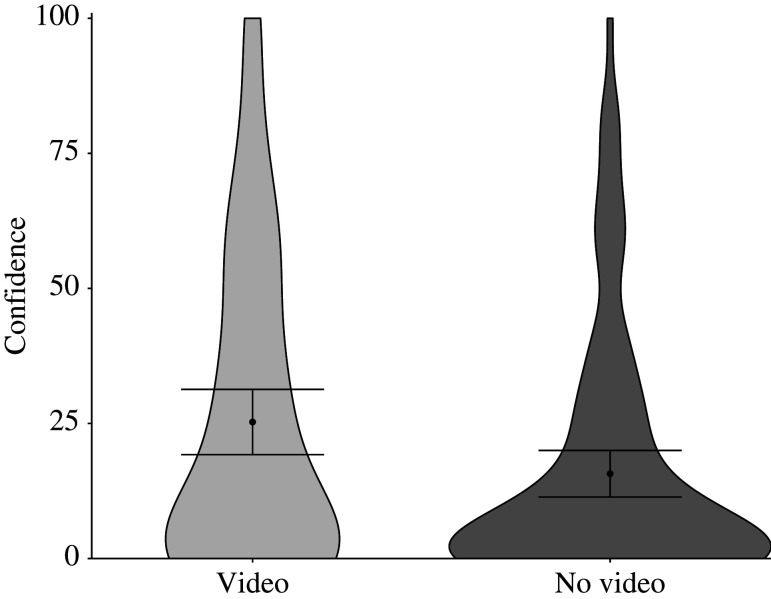
Subjects’ confidence ratings for the higher standard ‘as well as a pilot could’ question by condition: video, no video in Experiment 1. Error bars represent 95% confidence intervals of the cell means.

We then carried out the same analyses on subjects' confidence ratings for the higher standard, the ‘as well as a pilot could’ question. We display these data in figure 2. As the figure shows, we found the same pattern as with the ‘lower standard’ question. That is, subjects who watched the video were more confident than those who did not, *M*_video_ = 25.27, *M*_no video_ = 15.70, *M*_diff_ = 9.57, 95% CI [2.20, 16.95]. The Welch's t-test and Wilcoxon signed-rank test showed the same pattern, *t*_166_ = 2.56, *p* = 0.01; *Z* = 2.15, *p* = 0.03.

To determine if men and women reported different degrees of confidence, we first classified responses by gender and video condition; we then calculated mean confidence ratings. Two caveats are warranted. First, because there was only one non-binary subject, we excluded that subject from this particular analysis. Second, classifying data by gender broke random assignment. We checked to make sure there were similar numbers of men and women in each of the four cells; there were (refer to electronic supplementary material for more information). With these caveats in mind, we found that subjects who watched the video were more confident in their ability to land the plane both ‘without dying’ and ‘as well as a pilot could’ compared with subjects who did not watch the video. But we also found gender mattered for the ‘without dying’ confidence rating; men were more confident than women in every condition.

In other words, we conducted two separate (gender: men, women) x 2 (video: video, no video) ANOVAs, the first on subjects' ‘without dying’ ratings and the second on their ‘as well as a pilot could’ ratings. There was a main effect of video for both ratings, but also a main effect of gender for the ‘without dying’ rating, ‘without dying’ *M*_diff_ = 12.24, 95% CI [2.39, 22.10], *F*_1,196_ = 4.46, *p* = 0.01; ‘as well as a pilot could’; *M*_diff_ = 5.17, 95% CI [−2.34, 12.69], *F*_1,196_ = 1.96, *p* = 0.16. There was no interaction between gender and video condition for either measure (without dying: *F*_1,196_ = 0.85, *p* = 0.35; as well as a pilot could: *F*_1,196_ = 2.2, *p* = 0.14).

Next, as a preliminary investigation of mechanism, we determined the extent to which people who watched the pilot land the plane reported it was easier to imagine themselves landing a plane, relative to their counterparts who did not watch the pilot. We found no evidence to support this idea (*M*_video_ = 3.00, *M*_no video_ = 3.02; *M*_diff_ = −0.02, 95% CI [−0.42, 0.39], *p* = 0.93). But in unplanned follow-up analyses, we found people's ease of imagining and confidence they could land a plane without dying were strongly associated when they had watched the pilot (*r*_87_ = −0.57, 95% CI [−0.70, −0.41], *p* < 0.001) and weakly associated­—including plausibly zero—when they had not (*r*_107_ = −0.18, 95% CI [−0.36, 0.01], *p* = 0.06). A Fisher's z-test indicated that these correlations were significantly different (*r*_diff_ = −0.39, 95%CI [−0.61, −0.15], *z* = −3.18, *p* < 0.001). These findings fit with the idea that when people watched the pilot land the plane, they were more sensitive to ease of processing cues [38]. Moreover, the video might enable subjects to develop more detailed imaginations of themselves landing the plane, which they then misconstrue as confidence that they could actually execute the task.

Taken together, our findings show that watching one short non-instructional video of a pilot landing a plane inflated people's confidence in their own ability to land the plane—a specialized task requiring a great deal of expertise. One possible counter-explanation for our results could be that people do not know landing a plane requires much expertise, or that after watching the video, people re-evaluate their knowledge about what expertise is necessary. To address this possibility, we classified subjects according to whether they had watched the video and calculated their mean response to the question ‘How much expertise do you think is involved in landing a plane?’ (1 = *none*, 5 = *a great deal*). These means were approaching ceiling and nearly identical—findings that suggest people recognized that a great deal of expertise was necessary to land a plane, regardless of whether they watched the video, *M*_video_ = 4.39, *M*_no video_ = 4.36, *M*_diff_ = 0.03, 95% CI [−0.30, 0.37], *p* = 0.83. Considered together, these data do not support the counter-explanation.

The data from Experiment 1 provide preliminary evidence that increasing semantic context can inflate people's confidence in their own ability to perform a highly specialized task—in this case, landing a plane safely. In Experiment 2, we aimed to replicate these findings with a larger sample size to obtain a more precise estimate of the size of the effect [62]. We also aimed to address a weakness of Experiment 1: the fixed order in which we gathered data on the ‘without dying’ and ‘as well as a pilot could’ questions. In Experiment 2, we counterbalanced the order of these questions.

## 3. Experiment 2

We pre-registered this experiment. It, as well as materials and data, are available at https://researchbox.org/511. We report information not relevant to the central hypotheses in the electronic supplementary material.

### 3.1. Method

#### 3.1.1. Subjects

We recruited subjects from Amazon's Mechanical Turk (MTurk), an online source of a vast and diverse population, who received $0.30 USD for participating [57]. We used the Shiny Web ‘power for two independent groups t-test’ app (https://designingexperiments.com/shiny-r-web-apps/) to calculate sample size based on the data from Experiment 1. Using those data and a desired power 0.90, the target *n* per condition was 226, or a total of 502. Anticipating exclusions, we aimed to collect data from 600 people, but because Qualtrics and MTurk interact in such way that it is possible to unintentionally collect more data than required, we ultimately collected data from a total of 632 subjects. After applying exclusions as detailed below, we retained 582 subjects in the dataset (*M*_age_ = 39.19, s.d._age_ = 12.95; 31% identified as men, 68% identified as women and 1% identified as gender diverse); 94% of subjects reported English was their first language.

#### 3.1.2. Design

We used the same design as in Experiment 1, a between-subjects design with two conditions (video: video, no video).

#### 3.1.3. Procedure

We used the same basic method as in Experiment 1. This time, however, we counterbalanced the order of the ‘without dying’ and ‘as well as a pilot could’ questions.

### 3.2. Results and discussion

Consistent with our pre-registration, we first excluded 50 subjects who provided non-sensical descriptions of the situation they were asked to imagine, failed our attention check, had a valid pilot's licence or had flown or landed a plane before. We pre-registered an independent t-test with video (video, no video) as a between-subjects factor, collapsing the order of the dying and pilot questions, because we did not expect to see an effect for order. When we carried out this pre-registered test, we found subjects who watched the video of a pilot landing a plane were more confident in their own ability to land the plane at the lower standard, ‘without dying,’ *M*_video_ = 34.23, *M*_no video_ = 28.79, *M*_diff_ = 5.44, 95% CI [0.85, 10.03], *t*_561.43_ = 2.33, *p* = 0.020. But we did not find the same pattern for the higher standard, ‘as well as a pilot could’, *M*_video_ = 22.42, *M*_no video_ = 19.20, *M*_diff_ = 3.22, 95% CI [−0.97, 7.42], *t*_568.18_ = 1.51, *p* = 0.13.

**Figure 3.**
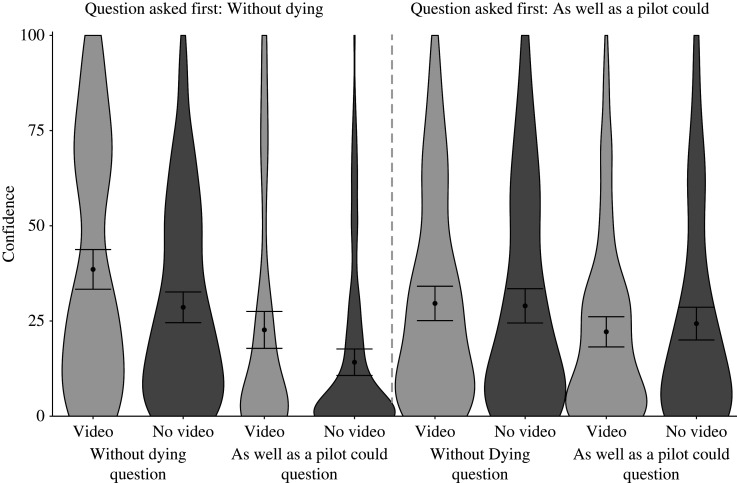
Subjects’ responses to the lower standard ‘without dying’ and higher standard ‘as well as a pilot could’ confidence questions split by condition (video, no video) and the order in which they were presented in Experiment 2. Error bars represent 95% confidence intervals of the cell means.

When we took a closer look with exploratory analyses, we found evidence that question order mattered (figure 3). That is, the left side of the figure shows that when subjects first saw the lower standard ‘without dying’ and then the higher standard ‘as well as a pilot could’ questions, the video boosted confidence on both. This condition was a replication of the method in Experiment 1. By contrast, the right side of the figure shows that when subjects first answered the higher standard and then the lower standard question, the video did not boost confidence on either measure (see also table 1).

**Table 1.** Confidence ratings by condition and question order for Experiments 1 and 2.

Exp	rating	without dying question asked first						as well as a pilot could question asked first					
		video			no video			video			no video		
		*Mdn*	*M*	95% CI	*Mdn*	*M*	95% CI	*Mdn*	*M*	95% CI	*Mdn*	*M*	95% CI
1	without dying	30	37.82	[31.66, 43.98]	21	29.50	[24.17, 34.83]	—	—	—	—	—	—
	as well as a pilot could	15	25.27	[19.24, 31.30]	5	15.75	[11.39, 20.11]	—	—	—	—	—	—
2	without dying	30	38.56	[33.35, 43.77]	20	28.60	[24.56, 32.63]	23	29.63	[25.11, 34.14]	20	28.99	[24.48, 33.49]
	as well as a pilot could	10	22.67	[17.82, 27.51]	3	14.18	[10.70, 17.65]	16.5	22.17	[18.20, 26.13]	15	24.32	[20.02, 28.63]

In null hypothesis testing terms, we conducted two separate 2 (question order: dying first, pilot first) × 2 (video condition: video, no video) ANOVA—the first on subjects' ‘without dying’ ratings and the second on their ‘as well as a pilot could’ ratings. For the ‘without dying’ ratings, we found a main effect of video condition, *F*_1,581_ = 5.23, *p* = 0.02; a marginal effect of question order, *F*_1,581_ = 3.40, *p* = 0.07; and an interaction, *F*_1,581_ = 4.04, *p* = 0.04, such that watching the video inflated people's confidence when the ‘without dying’ question was asked first, *M*_diff_ = 9.96, 95% CI [1.60, 18.31], *p* = 0.01; but not when the ‘as well as a pilot could’ question was asked first, *M*_diff_ = 0.64, 95% CI [−7.90, 9.17], *p* = 0.99. For the ‘as well as a pilot could’ ratings, we found a main effect of question order, *F*_1,581_ = 5.2, *p* = 0.02 and an interaction, *F*_1,581_ = 6.34, *p* = 0.01, such that watching the video inflated people's confidence when the ‘without dying’ question was asked first, *M*_diff_ = 8.49, 95% CI [0.87, 16.11], *p* = 0.02; but not when the ‘as well as a pilot could’ question was asked first, *M*_diff_ = −2.15, 95% CI [−9.93,5.63], *p* = 0.89

The data showed small deviations from normality and variance. But when Blanca *et al*. carried out a series of Monte Carlo simulations with a range of distributions including extreme skewness and kurtosis values (of up to skewness = 2 and kurtosis = 6), they found that ANOVA can be robust to such deviations [63]. Our skewness and kurtosis values were within these limits. Nonetheless, we also conducted a second analysis, a robust ANOVA [64]. Using this approach, we found similar results as with the original ANOVA. More specifically, subjects who watched the video of a pilot landing a plane were more confident that they could land the plane without dying than people who did not; *F*_1,581_ = 6.44, *p* = 0.01. The order of the questions mattered such that people were more confident they could land the plane without dying when they were asked that question first; *F*_1,578_ = 5.02, *p* = 0.03. We also found a marginally significant interaction; *F*_1,581_ = 3.53, *p* = 0.06, such that watching the video inflated people's confidence when the ‘without dying’ question was asked first.

Though these patterns are the result of exploratory analyses, they raise the possibility that responses to the first question anchored responses to the second question—in particular, being asked the lower standard ‘without dying’ question first allows subjects to nod along with feelings of ease they experience through watching the video, and although the second question about the pilot standard might shift subjects away from their intuitive hunch, they adjust insufficiently from their answer to the ‘without dying’ standard [59,65]. We could recast this idea in ‘System 1 versus System 2’ language by saying that when people considered their ability to perform the skill to a low standard, they took a System 1 heuristic approach, nodding along with recent feelings of easier mental simulation. But when other people compared themselves with a pilot, they took a System 2 systematic approach, because the comparison highlighted the expertise involved in performing the task. The System 1–2 distinction is broadly consistent with other approaches such as the Source Monitoring Framework.

Next, and consistent with our pre-registration, we determined if men and women reported different degrees of confidence. To address this issue, we again classified responses by gender and video condition, and calculated the mean confidence ratings. Like Experiment 1, we excluded the small number of non-binary subjects from this particular analysis (*N* = 6). We found that subjects who watched the video were more confident in their ability to land the plane ‘without dying’ compared with subjects who did not watch the video. But we also found gender mattered; men were more confident than women in every condition.

In other words, we conducted two separate 2 (video: video, no video) x 2 (gender: men, women) ANOVAs, the first on subjects ‘without dying’ ratings and the second on their ‘as well as a pilot could’ ratings. We again found a main effect of video, but also a main effect of gender (without dying: *M*_diff_ = 10.53, 95% CI [5.84, 15.75], *F*_1,575_ = 17.54, *p* < 0.001; as well as a pilot could: *M*_diff_ = 8.34, 95% CI [3.88, 13.03], *F*_1,575_ = 12.93, *p* < 0.001). There was no interaction between gender and video condition for either measure (without dying: *F*_1,575_ = 1.35, *p* = 0.25; as well as a pilot could: *F*_1,575_ = 0.31, *p* = 0.58). Of course, these data are exploratory, not randomized, and we therefore urge caution in interpretation.

Then, and also as a pre-registered analysis, we determined if people who watched the pilot land the plane reported it was easier to imagine landing a plane relative to their counterparts who did not watch the pilot. As in Experiment 1, we found no evidence to support this idea when comparing mean responses to this question across the video and no video conditions (*M*_video_ = 3.36, *M*_no video_ = 3.26; M_diff_ = 0.11, 95% CI [−0.13, 0.34], *p* = 0.11). But, consistent with the pattern of results from Experiment 1, we found that people's ease of imagining and confidence they could land a plane without dying was moderately associated when they had watched the pilot (*r*_279_ = 0.43, 95% CI [−0.52, −0.33], *p* < 0.001) and weakly associated­ when they had not (*r*_299_ = 0.26, 95% CI [−0.37, −0.15], *p* < 0.001). A Fisher's z-test indicated that these correlations were significantly different *(r*_diff_ *=* −0.17, 95% CI [−0.31, −0.02], *z* = −2.27, *p* = 0.02). These findings provide more support for the idea that the video may enable subjects to develop more detailed imaginations of themselves landing the plane, that they misconstrue as evidence that they could actually land the plane.

Finally—and as in Experiment 1—we found that regardless of whether subjects saw the video, they reported similar awareness that being a pilot demands great expertise (*M*_video_ = 4.50, *M*_no video_ = 4.49, *M*_diff_ = 0.01, 95% CI [−0.29, 0.31], *p* = 0.94).

Considered together, these findings show that when people watch a video of a pilot land a plane, they become more confident they could land a plane without dying. This finding replicates the major finding in Experiment 1. We also replicated the finding that men were more confident than women, and that people retained awareness of the fact that landing a plane requires expertise. Finally, our data add to the literature showing that when items tap into what people know, the order in which those items appear changes how they evaluate their prior performance [66]. Here, we extend this literature with data suggesting that when questions tap into what skills people think they have (but do not), the order in which those questions appear changes how they evaluate their future performance.

## 4. Mini meta-analysis

**Figure 4.**
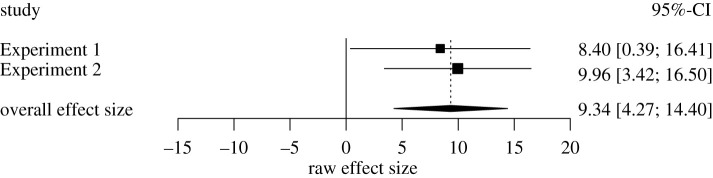
Mini meta-analysis of subject's confidence ratings by condition (R code; Carter & McCullough, 2014 [67]).

To obtain a more precise estimate of the effect of watching a video on confidence in one's ability to perform a complex task, we conducted a mini meta-analysis of the data from Experiment 1 and the subset of the data from Experiment 2 that followed the same method [62], and report those results in figure 4.

The right side of the vertical line in figure 4 shows an effect in which the video made subjects more confident in their ability to land a plane without dying. As the figure shows, subjects who watched the video were more confident in their own ability to land the plane than subjects who did not. That is, we found a weighted raw effect size of *M*_diff_ = 9.34 [4.27, 14.40], *p* < 0.01 or a 9.34% shift towards overconfidence. These findings fit with the idea that watching a short video of an expert performing a highly specialized task inflates people's confidence they too could perform that task.

How are we to understand this effect size? We can address this question by first turning to the related literature. First, our effect size is similar to that of the effect called **‘**truthiness,’ whereby photos boost the perceived truth of false claims—a mechanism thought to contribute to the ‘fake news’ problem [68]. Second, our 9% shift in overconfidence is similar to that demonstrated in a recent study on ‘imagination inflation’, whereby imagining a hypothetical event increased people's certainty that the event really happened. This mechanism is thought to contribute to problems associated with recovered memory therapy [69]. Third, our effect size is similar to a related illusion in which people who watched a video of a magician performing the tablecloth trick were more confident in their success actually performing the trick than people who merely thought about performing the trick but did not watch the video [28]. Fourth, our effect size is similar to another effect reported in the same paper in which people who watched a video of an expert throwing a dart at a bullseye, 20 times in a row, predicted they would score approximately 10 more points if they actually then threw a dart than people who watched the same expert video only once. This finding in particular highlights the pernicious effect of exposing people to multiple—but trivially informative—demonstrations of an expert performing a skill [28]. And finally, if we look to the wider literature, our effect size expressed as a correlation (*r* = 0.124) is reminiscent of the effect of antihistamine reducing runny noses, the link between combat exposure in Vietnam and PTSD over the next two decades, and the link between exposure to lead and impaired intelligence scores in children [70].

## 5. General discussion

We found evidence that simply watching one non-instructional demonstration of an expert performing a highly complex skill leads people to become more confident in their ability to perform that skill. More specifically, when people watched a trivially informative video of a pilot landing a plane, it inflated their confidence that they themselves could land a plane.

These findings contribute to several literatures. First, our findings suggest that increased semantic context creates illusions not just of prior experience, knowing or understanding—but also of the ability to actually do something implausible [26,35,71,72]. Our findings also extend prior work showing that increased semantic context leads people to predict rosy outcomes in the future. In one series of experiments, people evaluated positive or negative claims about the price of commodities in the future, such as ‘manganese is likely to have increased [decreased] in price three months from today’ [36]. Sometimes those claims appeared with a photo of the commodity; other times the claim appeared alone. Across those experiments, photos promoted rosiness about the future events, leading people to believe positive claims about the future but not negative claims. Here, we show that watching an expert perform a highly specialized task promotes people's rosiness in their ability to do something they almost certainly could not do.

Second, our findings fit with the literature suggesting that when imagining an experience in the future brings those self-generated mental products to mind easily—and with low cognitive markers of effort—people think that experience is more likely to happen [36,73]. Take, for example, one recent study in which people came to think there was a greater risk of experiencing climate change-related events when they conjured up easy-to-imagine hypothetical scenarios, such as the roads flooding while they were driving, than difficult-to-imagine ones, such as swimming in a lake in which soaring temperatures have promoted the growth of dangerous bacteria [74]. Our exploratory analyses provide preliminary evidence for a similar mechanism: when people watched the pilot land the plane, there was a strong association between how easy they found it to imagine landing the plane and how confident they were they could land the plane without dying. When people did not watch the pilot land the plane, there was plausibly no association between these measures. These findings provide tentative support for the idea that the video enables subjects to develop more detailed imaginations of themselves landing the plane, that they misconstrue as evidence that they could actually land the plane. But these analyses were exploratory, and such a conclusion is purely speculative. Considered together, these findings suggest increased semantic context not only increases people's estimates of the likelihood of future experiences, but it can also inflate their confidence in their abilities.

Third, these findings extend what we know about the kinds of situations in which people experience ‘knowledge neglect’—the failure to retrieve or apply their previously demonstrated knowledge, resulting in a failure to spot errors [55,75]. Here, we provide evidence that people also fail to retrieve or apply their demonstrated knowledge about the expertise involved in executing a complex skill when evaluating their own ability to execute that skill. Such a possibility fits with the idea that knowledge neglect is exacerbated when decisions are susceptible to fluency-driven biases [76]. Our findings suggest that when subjects first considered the emergency scenario and then watched the pilot landing a plane, knowledge neglect could have arisen from greater transportation into the scenario and easily accessible feelings of processing ease that people misattribute to their abilities.

Fourth, they fit with an anchoring-and-adjustment account in which the order of items affects people's evaluations of their performance on those items. That is, our findings from Experiment 2 support the possibility that the question people encountered first anchored their response to the second. In other words, when people first face the lower standard ‘without dying’ question, they might be lulled into a more heuristic type of processing, essentially ‘nodding along’ with the scenario, and tending to rely on recent feelings of easy processing that the added semantic context of the video provided [77]. Then, to the extent people have difficulty sufficiently adjusting away from their overconfidence, when they face the higher standard question—comparing themselves with a pilot—they report more confidence than people who did not watch the video. But what happens to people who encounter the questions in the opposite order? When they are asked to compare their ability with that of a pilot, the obvious high standard should make it difficult to nod along; when they are then asked about their ability in the lesser standard, they too adjust away from their low confidence—but never get to the point where they nod along. These are speculative mechanisms, and although they fit with the literature, more research is needed.

Although we cannot determine the extent to which these mechanisms—in whole or in part—drive our effects, across two experiments our data suggest a general mechanism in which the semantic context of the video helps people mentally manufacture thoughts, images and feelings consistent with the proposition that they could land the plane. But there is more work to be done. For instance, it is not clear exactly what aspects of the video led people to be overconfident. For instance, our video depicted a successful, smooth, landing with no complications. Perhaps if people saw a challenging landing, it might draw their attention to their genuine lack of skill. Furthermore, the video was not designed to be instructional (recall that an experienced commercial pilot who has trained many pilots pronounced it ‘useless’). Perhaps if the pilot in the video stepped through the specific actions necessary to land the plane, explaining the purpose behind them, that shift to a more concrete level of construal would encourage people to evaluate their abilities more accurately [78]. We know that people's confidence in their knowledge of complicated processes decreases when they are asked to provide a step-by-step explanation of how the process works [79]. For instance, when people considered their understanding of how a zipper works, they reported greater understanding than when they were asked the question at a more concrete level, such as how the parts of a zipper enable it to work [78]. Likewise, the relative lack of concrete steps in the video may have inflated people's confidence. On the flip side, to the extent prior work on truthiness illuminates some of the mechanisms driving our effects, it might be fruitful to introduce another condition in which some people saw an unrelated video. In the truthiness literature, people tend to classify more difficult trivia statements as false when those statements were paired with a semantically unrelated photo—compared with a related photo or no photo at all [72]. These findings fit with the idea that the lack of semantic relatedness in the unrelated photos produced disfluent processing.

In addition, the successful, smooth landing with no complications left subjects in the dark about the decisions driving the pilot's actions; subjects might therefore have developed their own error-filled notions to fill in the gaps. Such a process could be explained by the ‘beginner's bubble’ [80]. The idea is that when novices learn just a small amount about a complex task, their confidence in their performance disproportionately shoots up compared with their objective performance. The proposed mechanism for this bubble of overconfidence is that people develop incomplete or insufficient theories about how to perform the task, which in turn leads to rogue feelings of competence. Although our video was not intended to be instructional in any way, the fact that we chose a highly specialized task with which people had no prior learning makes it reasonable to speculate that people might have developed incomplete or insufficient ideas about how to land a plane.

Even though our findings fit with the idea that the video provided semantic context that supported fluent generation of relevant thoughts, images and feelings, it is possible that some mechanisms other than fluency have driven these effects. For instance, it is possible that our effects arise from the combination of knowledge neglect and the beginner's bubble. It is also worth considering the role of individual differences given that most people reported realistic appraisals of their ability to land a plane. First, the literature on the need for self-enhancement and narcissism suggests it can lead to ‘overclaiming’, or claiming to know terms that do not exist. Perhaps people high on these traits would be more susceptible to the illusions of confidence we report here [3,4]. Second, the literature on imagination inflation suggests that people who are high on hypnotic suggestibility and dissociativity are prone to developing false beliefs they have experienced an event after imagining it. It is possible people high on these traits would be prone to the illusory confidence-boosting effects of semantic context [81]. This possibility fits with our findings, in both experiments, that people who found it easier to imagine the situation were more confident in their ability to land a plane. Third, work on the illusory truth effect suggests that people are more susceptible to fluency biases when they are high on the need for cognition; the idea is that elaborative thought enhances processing and boosts familiarity at a later test [68]. Considered together, these possibilities suggest that the role of individual differences in moderating our effects is a topic worthy of future research.

The effects we report here might also be considered new and surprising examples of the confidence-boosting effects of videos demonstrated by Kardas & O'Brien [28], who found that when people watched (say) the same video, 20 times, of a man throwing a dart into the bullseye, they were more confident they themselves could successfully throw a dart closer to the bullseye than people who saw the video only once. We set out to test the boundaries of such an effect. We thought that landing a plane would pose as a reasonable boundary for two main reasons. First, it is a highly specialized skill requiring years of expertise. Second, the consequences of failing to perform such a task successfully could have disastrous consequences. Our findings suggest that this phenomenon holds for even highly specialized skills and further our understanding about the kinds of situations that might encourage people to be overconfident. Our hope is that future research will adopt and refine our method to explore and unearth its underlying mechanisms.

## Data Availability

Data, materials and electronic supplementary information are available at https://researchbox.org/511. The data are provided in the electronic supplementary material [54].
